# Areas with evidence of equity and their progress on mortality from tuberculosis in an endemic municipality of southeast Brazil

**DOI:** 10.1186/s40249-017-0348-5

**Published:** 2017-10-12

**Authors:** Mellina YAMAMURA, Marcelino SANTOS NETO, Francisco CHIARAVALLOTI NETO, Luiz Henrique ARROYO, Antônio Carlos Vieira RAMOS, Ana Angélica Rêgo de QUEIROZ, Aylana de Souza BELCHIOR, Danielle Talita dos SANTOS, Juliane de Almeida CRISPIM, Ione Carvalho PINTO, Severina Alice da Costa UCHÔA, Regina Célia FIORATI, Ricardo Alexandre ARCÊNCIO

**Affiliations:** 1 School of Nursing of the University of São Paulo, Ribeirão Preto Campus (EERP/USP), Ribeirão reto, SP Brazil; 2 Federal University of Maranhão (UFMA), São Luís, MA Brazil; 3 School of Public Health of the University of São Paulo (FSP/USP), São Paulo, SP Brazil; 4 Federal University of Rio Grande do Norte (UFRN), Natal, RN Brazil; 5 School of Medicine of the University of São Paulo, Ribeirão Preto Campus (FMRP/USP), Ribeirão Preto, SP Brazil

**Keywords:** Tuberculosis, Spatial analysis, Mortality, Cause of death

## Abstract

**Background:**

In Brazil, people still fall ill and die from tuberculosis (TB), and this can be explained by the significant impasse in the equity of distribution of therapeutic resources to the population as a whole. The aim was to identify geographical areas which have shown progress in terms of equity (of income, schooling and urban occupancy) and test its effect on mortality from TB in a municipality of southeast Brazil.

**Methods:**

It is an ecological study considering TB as the basic cause for deaths registered between 2006 and 2013 on the Mortality Information System and other variables obtained through the Demographic Census of the Brazilian Institute of Geography and Statistics (2010). The geographical area for analysis comprised the areas of coverage of the health services. Social indicators have been constructed through the Principal Component Analysis (PCA). The cases were geocoded and the annual mortality rate from TB was calculated with smoothing using the local empirical Bayesian method. Multiple linear regression was then performed. There was confirmation of the existence of spatial dependence of residue through the application of the Global Moran *I* test, and application of the Models with Global Spatial Effects, to identify the best standard of spatial regression.

**Results:**

The mortality rates ranged from 0.00 to 2.8 deaths per 100,000 people, per year. In the PCA, three indicators were constructed, and designated as indicators of income, social inequality, and social equity. In multiple linear regression, the indicator of social equity was statistically significant (*P* < 0.0001) but had a negative association, an adjusted R^2^ of 28.36% and with spatial dependence (Moran *I* = 0.21, *P* = 0.003455). The best model to deal with existing spatial dependence was the Spatial Lag Model.

**Conclusions:**

The better social conditions have shown progress in reducing mortality from TB, thereby reinforcing the achievement of Sustainable Development Goals. In addition, cartography was also applied, which can be replicated in other scenarios throughout the world, using a scope distinct from that of works traditionally produced in that it places the emphasis on social equity.

**Electronic supplementary material:**

The online version of this article (doi:10.1186/s40249-017-0348-5) contains supplementary material, which is available to authorized users.

## Multilingual abtracts

Please see Additional file 1 for translations of the abstracts into six official working languages of the United Nations.

## Background

Despite the general decline in the occurrence of tuberculosis (TB) and the mortality thereof, which is, indeed, a global phenomenon [1], the fact is that Brazil has, over the last ten years, shown a very feeble rate of decline in the number of cases, amounting to just 2 % per year. The *End TB* strategy has recently been published, and recommends a 95% reduction in mortalities from TB by the year 2035. However, at Brazil’s current snail’s pace, it is highly unlikely that the target will be reached in time, unless there is a significant transformation in the method of tackling the social and health-related causative factors [2, 3].

Regarding the medullary elements of mortality resulting from TB, the specialized literature mentions clinical and individual factors, including multidrug-resistant TB, the human immunodeficiency virus and other comorbidities [4]; risk-presenting habits or behavior patterns, such as the use of illicit drugs [5], smoking [6], consumption of alcoholic beverages [7]; social stigma; and the very organization of the health services, which have not operated based on the logic of equity, thereby failing to allocate quality services to the areas that need them most [8, 9].

From an ecological perspective, studies show that population groups that reside in economically deprived areas, without quality health services, also tend to show greater health problems, more sequelae, lower life expectancy, and worse prognoses [10]. In spite of some assumptions regarding the impact of social inequality on mortality from TB [11–13], very few are the studies that have sought to check this association or effect [9], very few studies on TB mortality have sought to evaluate this association or effect using a spatial approach [9].

The study carried out by Alvarez et al. [9] identified the impact of social iniquities on the mortality from TB, but this study was conducted in European countries. In contrast, in Latin America only one study has been carried out with this specific objective [14]. This means that, even though the *End TB* strategy has been emphatic with regard to social protection, through the assumption of a relationship between social factors and mortality from TB, according to some studies [15, 16], it is relevant to highlight the evidence of this association.

In general, studies about TB and equity/inequality [9, 15, 16] have aimed to measure casual relationships between these variable, they have failed in considering an appropriate statistical analysis when there is a spatial dependency. It is important to consider that measured geographic variables such as TB mortality may exhibit properties of spatial dependency, which means that the tendency of the same variables measured in locations in close proximity to be correlated, (it is not a independent event) and also there is a spatial heterogeneity (non-stationarity of most processes, meaning that global parameters do not exactly express processes occurring at a particular area). While traditional studies have treated two last features as a problem [9], studies with spatial approach could consider them precisely and explicitly.

Equity has been understood as the lack of potentially curable systematic differences (remediables) in one or more aspects of healthcare in population groups or subgroups that are socially, economically, demographically or geographically defined [17].

In the light of the new challenges presented by the End TB strategy and the many proposals that have been put forward to end poverty, assess social programs, promote equity and social well-being, while at the same time protecting socially disadvantaged sections of the population and their respective environments [18], this study aimed to analyze the relation between social equity (of income, schooling, and urban occupancy) and TB mortality rate through a spatial approach.

## Methods

### Study design and setting

This is an analytical ecological study [19], carried out in a Brazilian municipality which lies in the countryside of São Paulo State, lying 314 km from the capital of the state and 697 km from Brasília, at coordinates of 21° 10′ 36″ south latitude and 47° 49′ 15″ west longitude, at 546 m above sea level. The municipality has a total land area of approximately 650 km^2^, with a demographic density of some 981.3 people per km^2^ and an estimated population of 666,323 inhabitants in 2016, of whom 99.7% live in urban areas [20].

**Fig. 1:**
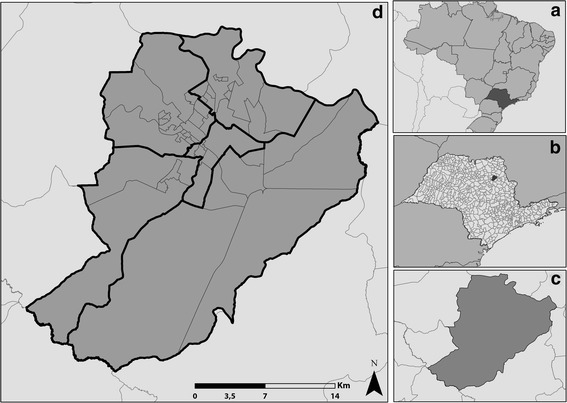
Map of the municipality under study and its geographical location. Ribeirão Preto – São Paulo, Brazil. Part (**a**) Brazil. Part (**b**) State of São Paulo. Part (**c**) Ribeirão Preto. Part (**d**)  Health district,  Areas of coverag,  Municipalities of SP – Ribeirão Preto. Source: Adapted from geographical bases. IBGE [20] and the Municipal Secretariat of Health of the city of Ribeirão Preto

Regarding the geo-politico-administrative organization of the basic healthcare networks, the municipality is divided into five Health Districts and 46 areas of catchment of Primary Health Care (PHC) (Fig. 1). With regard to the epidemiological indicators for TB, until 2007 Ribeirão Preto was considered a priority municipality for actions to control the disease, due to the high occurrence of cases. This same year, the rate was 28.7 cases per 100,000 people. Today, this municipality is not considered as a priority area; however, in 2014 the rate of prevalence of TB was similar to that of 2007, with 28.5 cases per 100,000 people [21].

### Study population and operational criteria

The population of the study consisted of cases of deaths from TB as registered on the Mortality Information System (MIS) between 2006 and 2012. The data was collected in May 2013, at the Epidemiological Surveillance Division of the Municipal Secretariat for Health in the city of Ribeirão Preto, State of São Paulo, Brazil. In this procedure, we considered the Death Certificate (DC) of people living in the urban part of the municipality, whose main cause of death was assigned an International Classification of Diseases, Tenth Edition (ICD-10) code between A15.0 and A19.9, which includes all clinical forms of TB.

### Data analysis

Initially, the demographic variables (sex, age, ethnicity/colour, marital status, schooling and occupation) and also the clinical/operational variables (place of death, medical assistance, necropsy, clinical format, and medical professional responsible for filling in the DC), were analyzed for completeness and consistency. The next step was descriptive analysis, including the calculation of absolute and relative frequencies of the categorical variables. For the age variable, statistical measures of position were obtained (mean, median, maximum and minimum values), as was the standard deviation, as a measure of dispersion. Later, this variable was dichotomized according to the median. These variables were not included in the statistical modeling stage.

For the construction of equity indicators, we selected variables from the 2010 Demographic Census of the Brazilian Institute of Geography and Statistics [22], namely: nominal income per head; literacy level of the person responsible for the family; the gender of the person responsible; and the number of people living in the household. The technique of Principal Component Analysis (PCA) consists of a linear combination to select the most representative variables in each indicator or component that is to be constructed. This technique may not only obtain the individual information of each component, but also the joint information of the most important component pairs in the analysis; The degree of importance is measured through the magnitude of the variance explained in the data set, usually above 70% [23, 24].

Another criterion for selecting the equity index used was the Kaiser method, which considers eigenvalues above one as relevant, since they generate components with a significant amount of information from the original variables [23]. The PCA analysis was conducted using the Statistica 12.0 software package.

**Fig. 2:**
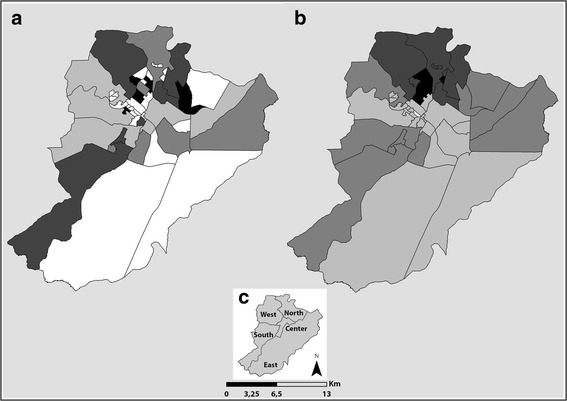
Distribution of mortality before and after using the local empirical Bayesian method. Part (**a**) Gross mortality rate from tuberculosis per 100,000 people.  0.0,  0.01–0.89,  0.90–2.07,  2.08–2.71,  2.72–5.93. Part (**b**) Bayesian mortality rate from tuberculosis per 100,000 people.  0.0,  0.01–0.83,  0.84–1.67,  1.68–2.04,  2.05–2.82. Part (**c**) Health District

After the verification of the characteristics of the construct, in terms of composition (variance) of the original variables, the scores were calculated, for the classification of the areas under study, based on quartiles. Considering the structure of the organization of health services in the scenario under study, the geographical area for analysis was defined as being the areas of coverage of the health services, close to the patients’ places of residence, known as PHC (Fig. 2).

The geographical encoding of the deaths from TB was carried out using Terraview 4.2.2 software, with the use of the digital map of the segment of streets, StreetBase®, for the city of Ribeirão Preto, with a projection Latlong/WGS84, acquired through the company Imagem Soluções de Inteligência Geográfica. The final geocoded data file followed Mercator’s Universal Transverse Projection and datum Geocentric Reference System for the Americas 2000.

Then, there was an analysis of the area considering the total deaths in each territory and its respective population, for the calculation of the annual mortality rate from TB. Being aware that the mortality rates of TB in the different areas were subject to the so-called oscillations of small figures, it was decided to implement the smoothing process provided by the use of the local empirical Bayesian model, which smooths the gross rates by applying weighted averages, resulting in a corrected and more stable rate, considering not only the information of the area, but also those of its neighborhood. At this stage, we used the Terraview 4.2.2 and ArcGis 10.2 software packages for the preparation of the chloropeth maps.

Defining the dependent (Bayesian mortality rate from TB) and independent variables (evidence of equity), there was the drawing up of the rook-type contiguity spatial weight matrix, using the software package OpenGeoDa 1.0. This type of matrix considers only neighborhoods that have common boundaries. Using the R 3.0.2 software package, there was a modeling of multiple linear regression, using the method of least squares, based on the criterion of choosing the best explicative model and the highest adjusted R^2^ value. After choosing the model for linear regression, the residue was analyzed to check the presence of spatial dependence, through the application of the Moran Global I Test [25]. It was verified if residues presented normal distribution, zero mean, constant and independent variance, indicating homoscedasticity [26].

If there is any spatial dependence in the errors of the individual observations within the model for linear regression, this goes against the assumption that these should be independent, in which case it is necessary to use a type of modeling that considers spatial self-correlation. In this case, there was the application of the models with global spatial effects, which considers the special correlation structure in a parameter before including it in the regression model, so as to identify the best model of spatial regression to be used, these being the spatial lag model (SLM), which assigns spatial self-correlation to the dependent variable, or the spatial error mode (SEM) which assigns the self-correlation to the mistake. The definition of the best model was based on the comparison of the lowest value within the Akaike information criterion (AIC), which establishes the maximum value of the logarithm of the probability, and the number of parameters of the model [27].

## Results

Between 2006 and 2012, we identified 50 deaths that had TB as their main cause. In Table 1, we can see the social and demographic profiles of the cases of deaths from TB. We observed a prevalence of the male sex (*n* = 41; 82%), Caucasian ethnicity (*n* = 35, 70.00%), marital status (*n* = 19, 38.00%), with schooling and occupation ignored or left blank, which respectively accounted for *n* = 33, 66.00%, and *n* = 27, 54.00%. With regard to age, we observed a mean age of 61 years (minimum age of 34 years and maximum of 89).

**Table 1:** Sociodemographic, clinical and operational characteristics of the deaths with TB as their main cause

Variables	Number	Percent
Sociodemographic characteristics		
Gender		
Male	41	82
Female	09	18
Age		
65 years and over	27	54
Under 65	23	46
Ethnicity/colour		
White	35	70
Black	11	22
Oriental	01	2
Left blank	03	6
Marital status		
Single	15	30
Married	19	38
Divorced	03	6
Widowed	04	8
Ignored	09	18
Schooling		
None	02	4
4 to 7 years of study	12	24
8 to 11 years of study	03	6
Ignored	33	66
Occupation		
Retired/pensioner	15	30
Homemaker	02	4
Miscellaneous	06	12
Ignored	27	54
Clinical and operational characteristics		
Place of death		
Hospital	36	72
Other healthcare establishment	10	20
At home	03	6
Others	01	2
Received medical assistance		
Yes	31	62
No	01	2
Ignored	18	36
Diagnosis confirmed by further examinations		
Yes	15	30
No	08	16
Ignored	27	54
Diagnosis confirmed by surgery		
Yes	07	14
No	22	44
Ignored	21	42
Diagnosis confirmed by necropsy		
Yes	27	54
No	19	38
Ignored	04	8
Clinical form of the disease by ICD-10		
A 15.2 Tuberculosis of lung, confirmed histologically	18	36
A 15.3 Tuberculosis of lung, confirmed by unspecified means	01	2
A 16.2 Tuberculosis of lung, without mention of bacteriological or histological confirmation	25	50
A 16.5 Tuberculous pleurisy, without mention of bacteriological or histological confirmation	01	2
A 17.8 Other tuberculosis of nervous system	01	2
A 18.0 Tuberculosis of bones and joints	01	2
18.8 Tuberculosis of other specified organs	01	2
A 19.8 Other miliary tuberculosis	01	2
Filling-in of certificate of death		
Assistant	12	24
Substitute	06	12
Death verification service	19	38
Others	11	22
Left blank	02	4

Regarding clinical and operational characteristics (Table 1), most of the cases were of the clinical pulmonary type (*n* = 45, 90.00%), and part of the cases (*n* = 27, 54.00%) were subjected to a necropsy to confirm the diagnosis regarding the main cause of death.

Regarding the application of the PHC for the construction of equity indicators, according to Table 2, we saw the establishment of three indicators of equity, with a total variance accounting for some 79.5% of the total universe of data. The first equity indicator had a variance of 46.21%; the second had 8.76%, and the third had 14.61%. The indicators with conditions that are more favourable to equity with regard to income, level of schooling and housing unit are shown with a plus sign (+), while those that are furthest away have a negative sign (−).

**Table 2:** Loads or eigenvectors for original variables for the construction of the indicators of equity

Original variables	Equity		
	Indicator 1 (+)	Indicator 2 (−)	Indicator 3 (++)
Proportion of households with a nominal monthly income per head between ½ and 1 national minimum wage	−0.39	0.15	−0.00
Proportion of households with a nominal monthly income per head between 1 and 2 national minimum wages	−0.40	0.12	−0.05
Proportion of households with a nominal monthly income per head between 2 and 3 national minimum wages	−0.22	0.22	0.52
Proportion of households with a nominal monthly income per head between 3 and 5 national minimum wages	0.35	0.15	0.33
Proportion of households with a nominal monthly income per head between 5 and 10 national minimum wages	0.41	−0.08	−0.00
Proportion of literate responsible females	−0.11	−0.55	0.17
Proportion of literate responsible males	−0.11	−0.51	0.34
Proportion of permanent private households with 3 people resident	−0.14	−0.37	0.31
Proportion of permanent private households with 5 people resident	−0.11	0.38	0.51
Proportion of permanent private households with 8 people resident	−0.39	0.06	−0.11
Proportion of permanent private households with ten or more people resident	−0.32	−0.11	−0.29

The first equity indicator considers more positive variables (+) with regard to the construct of equity, with a prevalence of people earning between 3 and 10 national minimum wages, and with a negative relationship considering households with 8 people or more. The second equity indicator considers more negative characteristics (−) with regard to the construct, with heads of household (men and women) who are not literate and who live with five or more residents. The third equity indicator considers variables that are closer to equity, with a prevalence of people earning between 2 and 5 minimum wages; literate people being responsible for the households, and households having between three residents and a maximum of five

Table 2 shows the characteristics of the equity construct as defined for this study, and in this table we can check the contribution of the weight of each original variable for the same, with contributions of more than 0.3 being mentioned in the table.

Figure 2 shows two maps, one with the gross mortality rates and the other with the rates duly smoothed with the application of the local empirical Bayesian method, with the mortality rates ranging from 0 to 2.8 cases per 100,000 people.

The smoothed mortality rate was then tested with regard to the three indicators of equity, and it was observed that the only indicator to be shown as statistically significant (*P* < 0.0001) was that of Indicator 3. However, this had a negative estimate and an adjusted R^2^ equivalent to 28.36%. In the appraisal of spatial dependence of residue through the Moran Global Test, a statistically significant value was obtained (Moran *I* = 0.21, *P* = 0.003455).

In the application of models with global spatial effects, SLM was the best option to deal with spatial dependence, having an AIC value lower than that of the model of linear regression and also producing residue without any spatial dependence.

Through this model, it was possible to confirm that social equity has had a negative association with mortality from TB, which means that each increase in this unit (equity) would be equivalent to an 8.8% reduction in the mortality rate (Table 3).

**Table 3:** Model of spatial regression for mortality rates for tuberculosis in the city of Ribeirão Preto

Intercept and components	Estimate	Standard error	*t*-value	*P*-value
Intercept	135.90	47.75	2.84	0.0044
Social Equity (CP3)	−8.8	19.26	−3.19	0.0013
Rho (*ρ*)	0.5316			0.0014

AIC: 606.07 (AIC linear regression: 614.26)

## Discussion

The study has sought to identify geographical areas that have shown progress in terms of equity (in terms of income, schooling, and urban occupancy) and also to test its effect on mortality rates from tuberculosis in a municipality in the southeast Brazilian region. It was observed that the mortality rate from TB in a certain area had been significantly affected by the rates in neighboring areas, which proved the need for a model for spatial regression which considers the spatial dependence of errors, and this showed that equity had a negative correlation to mortality from TB.

The study also showed that most deaths caused by TB occur in male patients, a fact that was already expected, as men at an active age are more often affected by the disease than women, as confirmed by a review of specialized literature, carried out by Neyrolles and Murci [28].

One worrying piece of data is that the most common clinical manifestation was the pulmonary form without any bacteriological or histological confirmation (ICD 16.2), but most of the cases were subjected to a necropsy which did in fact confirm the tardy diagnosis of TB. Parrechi and Ribeiro [29] say that, even though the process for the diagnosis of TB is actually quite simple, many of the cases are diagnosed in hospitals, which incurs high costs for the health systems and the possibility of sequelae for the patients [30]. In this regard, it becomes important to restructure and strengthen the actions for controlling TB through primary healthcare attention, with the prioritization of bacilloscopy of catarrh and also a rapid molecular test for the diagnosis, control and monitoring of the disease, well as the enhancement of the registration systems and the health information systems (HIS) [30].

Studies show that PHC still has some problems in the detection and the appropriate referral for the treatment of people with symptoms of TB. Together with these difficulties, there is the issue of social inequality, represented by a disorganization of services and teams, excessive bureaucratization of the process, between detection and referral for treatment; social and economic problems of the patients, which are not taken into consideration by the healthcare teams, and also prejudice and activities generating stigma, on the part of health professionals [31, 32].

The results also show that most of the patients were seen by a hospital prior to death. However, it was not possible to note whether the patient who eventually died was actually hospitalized during treatment, and what stage of treatment they were at, whether at the initial phase or at a more advanced stage thereof. A research study conducted in Ethiopia, also addressing the issue of death, showed that almost 75% of the deaths took place within the eight months of treatment, which is a cause for concern [33].

In a research study carried out by Liu et al. [34], it was observed that difficulties in the diagnosis of TB led to a delay in the treatment of over 40% of the subjects of the research study, and 69% of the patients with TB died before the completion of treatment. Similar data were found in a study carried out by Parhar et al. [35] in Alberta, Canada, in which 23% of the patients with TB died before starting treatment and 76.9% died before completing 60 days of therapy.

Regarding the equity indicators, the selection was based on the works of Sanches and Ciconelli [36] and Santos et al. [37] which consider income as being a determining factor for inequalities in health issues, as this variable is closely tied in with the issue of social well-being. The ways in which income affects health can be understood mainly through its use in the acquisition of health assets and services, access to health services, housing conditions, and education.

These authors [10, 38, 39] consider social justice as the equality of rights and opportunities, in the equalitarian distribution of resources and/or supplies, as the chance of survival of a child from a more affluent family is greater than that of a child from a family unit with lower purchasing power, with this social status being the most significant element in establishing a child’s general health conditions.

Regarding the findings of this study, there is a situation similar to that mentioned above, as the health districts of Ribeirão Preto with the higher mortality rates were the West and North regions, which are also the districts with greater social vulnerability [40, 41] and also those with greater incidence and prevalence of TB, as made evident in the study carried out by Hino et al. [42].

In addition, other authors [43] mention that areas that have serious shortcomings with regard to decent housing and basic sanitation also tend to try using health services that do not provide many solutions and which have limitations in terms of general supply and health services provided. In this regard, on analyzing the spatial relationship between deaths caused by TB and social indicators, it was possible to confirm that, whenever there is an increase in the degree of equity, there is also an important reduction in deaths caused by TB.

In addition, with the identification of the negative connection between social equity and deaths from TB, it becomes essential to point out that equity is a constitutional principle and should be an essential base for the production and supply of actions aimed at minority groups who are unprotected in terms of government policies, and that should be targeted by social policies. Indeed, this can be a decisive factor in the reduction in the number of deaths [44] and can therefore explain the results found.

In Brazil, as in other countries in Latin America, social policies have been implemented over the last two decades, favoring better rates of social equity, and this has shown a positive impact, bringing about an improvement in healthcare and in general well-being in these countries. Studies show that these social and intersectorial policies have led to an increase in the access to health services and also educational services; to devices and opportunities for work and income; to an improvement in the general health of the specific groups that have been analyzed; and also in the environmental area, with an increase in capital stock in communities and the empowerment of groups with an expansion of social participation and organization of civil society, not to mention general improvement in health for tackling disease and epidemics [45–47].

In this regard, the findings of the research study allow us to conclude that areas that have shown progress in public policies, with better distribution of income, quality of housing, and level of literacy of heads of household, including female heads of household, have also managed to achieve better levels of general health, with a reduction in the number of deaths due to TB.

Authors consider that, even though biological variations (gender and age) determine differences in morbidity and mortality, most health conditions are established through social factors rather than from natural variations internal to the individual [48]. This means that the groups with greater social vulnerability tend to suffer greater impact in terms of general health, thereby showing greater occurrence of the diseases and also experiencing more dramatic results as a result of this exposure. Evans et al. [48] acknowledge that this is the most important dimension within social equities, and the World Health Organization has highly recommended studies with this focus, in order to gain deeper understanding of the phenomenon, so as to achieve a certain balance between supply and needs.

This means that equity of territories is a goal to be achieved when seeking reduction of deaths from TB. It is also important to stress that equity does not mean the same as equality, as equity takes into account the fact that people are different and have differing needs. This means that if the same standard of service is given to all patients with TB, in the same way, in all places, there is the possibility that unnecessary action may be offered to some, while the needs of others are not met, thereby intensifying the situation of inequality.

There are groups with greater social vulnerability, and these should get priority in terms of supply of actions. This means that it is important to view equity as a process for the transformation of the scenario within which TB is present. The authors also consider that equity is not a rigid process, changing its coverage and scope as certain results are achieved, in the light of the dynamism of the population and of health needs [48].

A systematic review by Simwaka et al. [49] on the issue of equity and TB clearly showed the difficulty of local health systems, in operating with the distributive logistics of actions. It also showed that, throughout the chain of progression of TB (from the moment of falling ill, through to the completion of treatment) many cases have been lost as a result of the sensitivity of services to socially deprived minority groups. Another study, which also sought to identify equity, could confirm that areas where the people had a better level of schooling these also had greater access to services and, as a result, a better prognosis with regard to TB [8].

In terms of the situation of TB itself, it is important to point out that countries with a greater gross domestic product and lower inequality of distribution of income also have lower rates of prevalence of TB, due to the reduction of marginal effects. This means, in other words, that the greater the economic development of a country, the greater the availability of social investments as a way to promote health, education, and basic sanitation [50].

In general, studies show that intersectorial government policies, implemented in different parts of the world and placing an emphasis on social and economic aspects as important factors for the establishment of health, as well emphasizing social capital as a relevant factor for social cohesion which is necessary for the implementation of policies with the intention of reducing social and health inequities, have shown important progress in the improvement of levels of healthcare, increase in equity, and a rise in the general well-being of the population. Together with these, other studies also refer to actions seeking to improve the course of life, with the insertion of actions to promote health in vulnerable territories and groups, from pregnancy, with newborn babies being monitored throughout infancy, thereby reducing the rates of mortality and malnutrition among young children, in a move to prevent disease in adulthood and in old age [51–53].

Regarding limitations, we should mention the use of ecological delimitation, which has restrictions inherent in the method itself, and it is not always possible to infer the individual level from the findings at the level of the analysis unit used (coverage areas of health services) for the individual level [54], a view known as ecological fallacy [19].

On the other hand, this delimitation, differently from the epidemiological studies with an individual base, allows one to take into account variables related to context, this being useful for the investigation of the relationship between social and economic factors [55] such as equity and deaths from TB.

The ecological fallacy may be significantly reduced through the use of small analysis units and homogeneities. This brings a consequence which is the occurrence of excess zero, as death through TB is a rare event. In this study, the method that was found to reduce these factors was that of using, as the analysis unit, the areas of coverage of the health services, and local empirical Bayesian rates. These, on correcting the random fluctuation of mortality rates due to TB, reduce the number of units with zero rates, thereby making modeling feasible. The main limitation to this solution was the induction of spatial dependence on the mortality rate, something that was circumvented through the use of techniques of spatial regression. In the case of this study, both the initially unexplained spatial dependence and that induced through use of the Bayesian rates are represented, in this model, by the Rho (*ρ*) parameter (see Table 3), with the production of residue without spatial dependence.

Another limitation of the study is related to use of the PCA technique, that assume only linear relationships between attributes and do not test other relationships. The PCA is one of the simplest techniques in the field of multivariate analysis and has been widely used in epidemiological studies [56–58]. In addition, the study has considered secondary data from MIS, which means to work with ignored data, inexistent addresses and not fille. Another point to consider is the lack of an interface between the MIS and other HIS, which could open the floodgates to subnotification. However, it must be stressed that the use of these systems is extremely important, due to the possibility of analysis of data on a national scale, in a manner that is more or less homogeneous. In addition, the use of the MIS also allows the assessment of the quality of data in these systems, which often leads to the enhancement thereof.

For future studies, it would be interesting to conduct longitudinal monitoring of the patients afflicted with TB, to verify individual or clinical variables that could explain the deaths caused by the disease. Another interesting proposal would be that of having studies that include the PHC professionals in the areas of coverage, particularly in the areas where the occurrence of these events is most critical, so as to check the critical bottlenecks in terms of supply and organization of the services, for accessibility by the patient with TB.

## Conclusions

The study revealed the relationship between equity and mortality due to TB; in areas with greater social equity there was a reduction in the rates. The research was conducted using an important health information system (MIS), which still presents critical issues such as missing data and inexistent addresses, despite to be being an information system that is considered to be the gold standard in Brazil.

Since TB mortality is an event that is socially and spatially determined, it is imperative that researchers use robust and coherent methods to treat the spatial dependency. This cannot be ignored in the current studies concerned with this theme. The study brought relevant contributions to “End TB strategy”, when evidences that the areas with better levels of equity (better life conditions) have progressed more in terms of TB mortality than others, which is really important to define the publics policy and to advocate in favor of “social protection” as an action to face TB.

## Additional file

Additional file 1: Multilingual abstracts in the six official working languages of the United Nations. (PDF 752 kb)

## Abbreviations

AIC: Akaike information criterion
DC: Death Certificate
HIS: Health information systems
ICD-10: International Classification of Diseases- Tenth Edition
MIS: Mortality Information System
PCA: Principal Component Analysis
PHC: Primary Health Care
SEM: Spatial error model
SLM: Spatial lag model
TB: Tuberculosis
