# Supplementary material for: Areas with evidence of equity and their progress on mortality from tuberculosis in an endemic municipality of southeast Brazil
Source: Infect Dis Poverty. 2017 Oct 12;6:134. doi: 10.1186/s40249-017-0348-5 (PMC5637336; doi:10.1186/s40249-017-0348-5)
Supplement: Additional file 1: — Multilingual abstracts in the six official working languages of the United Nations. (PDF 752 kb) [file 40249_2017_348_MOESM1_ESM.pdf]

Translation of the abstract into the six official working languages of the United Nations

المناطق التي ثبت فيها تطبيق العدالة الاجتماعية وما أحرزته من تقدم فيما يتعلق بالوفيات الناجمة عن مرض التدرن (السل)، في بلدية مَوطونَة في جنوب شرق البرازيل.

ميلينا يامامورا، مارسيلينو سانتوس نيتو، فرانسيسكو تشيارافالوتي نيتو، لويس إنريكه أرويو، أنتونيو كارلوس فييرا راموس، أنا أنجليكا ريجو دي كيروز، أيلانا دي سوزا بيلشيور، دانييل تاليتا دوس سانتوس، جولين دي ألميدا كريسبيم، لون كارفاليو بينتو، سيفيرينا أليس دا كوستا أشا، ريجينا سيليا فيوراتي، ريكاردو ألكسندر آرسنسيو

ملخص

خلفية: لا يزال الناس يمرضون ويموتون بسبب مرض التدرن (السل) في البرازيل، ويعزى هذا المأزق الكبير إلى عدم الإنصاف في توزيع الموارد العلاجية للسكان ككل. وكان الهدف هو تحديد المناطق الجغرافية التي أظهرت تقدما من حيث الإنصاف من ناحية (الدخل والالتحاق بالمدارس والإشغال الحضري) واختبار أثره على معدلات الوفيات الناجمة عن مرض السل في بلدية في جنوب شرق البرازيل.

الأساليب: تعتبر الدراسة الإيكولوجية بأن السل هو السبب الرئيس للوفيات المسجلة بين عام 2006 وعام 2013 على "نظام معدلات الوفيات" والمتغيرات الأخرى التي تم الحصول عليها من خلال "التعداد السكاني" للمعهد البرازيلي للجغرافيا والإحصاءات (2010). وشملت المنطقة الجغرافية للتحليلات مجالات تغطية الخدمات الصحية. وقد تم بناء المؤشرات الاجتماعية من خلال تحليل المكونات الرئيسية (PCA). وقد تم ترميز تلك الحالات، وتم حساب معدل الوفيات السنوي من السل باستخدام طريقة بايزي التجريبية المحلية. ثم تم تنفيذ الانحدار الخطي المتعدد. وكان هناك تأكيد على وجود الاعتماد المكاني للبقايا من خلال تطبيق اختبار موران العالمي الأول، وتطبيق النماذج مع الآثار المكانية العالمية، لتحديد أفضل معيار الانحدار المكاني.

النتائج: تراوحت معدلات الوفيات بين 0.00 إلى 2.8 شخص لكل 100000 شخص في السنة الواحدة. وقد تم إنشاء ثلاثة مؤشرات خلال تحليل المكونات الرئيسية، وتم تصنيفها كمؤشرات للدخل، وعدم المساواة الاجتماعية، والعدالة الاجتماعية. كان مؤشر العدالة الاجتماعية ذو دلالة احصائية في الانحدار الخطي المتعدد (P<0.0001) وكان له ارتباط سلبي، وتعديل R^2 من 28.36٪ ومع الاعتماد المكاني (موران I = 0.21، P < 0.003455). وكان أفضل نموذج للتعامل مع الاعتماد المكاني القائم هو نموذج التجاوز المكاني.

الاستنتاجات: قد أظهرت أفضل الظروف الاجتماعية تقدما في خفض معدلات الوفيات من مرض السل، مما يعزز تحقيق "أهداف التنمية المستدامة". طبق رسم الخرائط أيضا بالإضافة إلى ذلك، حيث يمكن تكرارها في سيناريوهات أخرى في جميع أنحاء العالم، وذلك باستخدام نطاق يتميز عن الأعمال التي يتم إنتاجها تقليديا من حيث أنه يركز على العدالة الاجتماعية.

Translated from English version into Arabic by Mahmoud Sami, through

TRANSLATORS WITHOUT BORDERS

巴西东南部一个结核病流行市中具有公平性证据的地区及其在结核病死亡率方面的进展

Mellina Yamamura, Marcelino Santos Neto, Francisco Chiaravalloti Neto, Luiz Henrique Arroyo, Antônio Carlos Vieira Ramos, Ana Angélica Rêgo de Queiroz, Aylana de Souza Belchior, Danielle

Talita dos Santos, Juliane de Almeida Crispim, Ione Carvalho Pinto, Severina Alice da Costa Uchoa, Regina Célia Fiorati, Ricardo Alexandre Arcêncio

摘要

引言：在巴西人们仍会罹患结核病并致死，从整体来看这可以解释为全民医疗资源分配公平性方面的严重僵局。本研究的目的在于确定在公平性方面（包括收入、教育和城市入住率）已经取得进展的地理区域，并测试其对巴西东南部一市结核病死亡率的影响。

方法：将结核病作为 2006-2013 年间死亡率信息系统登记的基本死亡原因，和巴西地理统计研究所的人口调查（2010 年）获得的其他变量进行生态学研究。分析的地理区域由卫生服务的覆盖范围组成。通过主成分分析（PCA）建立了社会学指标。对案例进行地理编码，并使用局部经验贝叶斯方法(local empirical Bayesian method)计算 TB 的年死亡率。然后进行多元线性回归。通过应用 Global Moran I test 和全局空间效应模型确定了残留物的空间依赖性的存在，以确定空间回归的最佳标准。

结果：年均死亡率为 0-2.8/10 万。在主成分分析中，构建了 3 个指标，并指定为收入、社会不平等和社会公平的指标。在多元线性回归中，社会公平指标有统计学意义（$P<0.0001$），但呈负相关; 调整后的 R^2 为 28.36%，存在空间依赖性（Moran I＝0.21，$P=0.003455$）。处理现存的空间依赖的最佳模型是空间滞后模型。

结论：良好的社会条件表明在降低结核病死亡率方面取得了进展，从而促进了可持续发展目标的实现。此外，本研究还使用了制图学, 这可在世界各地的其他情况下应用，因其重点在于社会公平, 所以与传统意义上的应用范围有所不同。

Translated from English version into Chinese by Zhou Guan, edited by Pin Yang

CDC CHINESE CENTER FOR DISEASE CONTROL AND PREVENTION
NATIONAL INSTITUTE OF PARASITIC DISEASES

Zones d'inégalités et progrès vers l'équité en termes de mortalité due à la tuberculose dans une municipalité d'endémie du Sud-est du Brésil

Mellina Yamamura, Marcelino Santos Neto, Francisco Chiaravalloti Neto, Luiz Henrique Arroyo, Antônio Carlos Vieira Ramos, Ana Angélica Rêgo de Queiroz, Aylana de Souza Belchior, Danielle Talita dos Santos, Juliane de Almeida Crispim, Ione Carvalho Pinto, Severina Alice da Costa Uchoa , Regina Célia Fiorati, Ricardo Alexandre Arcêncio

Résumé

Contexte: On meurt encore de la tuberculose au Brésil, à cause notamment de l'important problème que pose l'équité de la distribution des ressources thérapeutiques dans la population générale. Notre but était d'identifier les régions ayant fait des progrès en termes d'équité (de revenus, de scolarisation, d'occupation urbaine) et de tester les effets de ces progrès sur la mortalité liée à la tuberculose dans une municipalité du sud-est du pays.

Méthodes: Il s'agit d'une étude écologique examinant la tuberculose comme cause principale de décès enregistrés entre 2006 et 2013 dans le Système d'informations sur la mortalité, ainsi que d'autres variables obtenues par le biais du recensement démographique de l'Institut brésilien de géographie et

de statistiques (2010). La zone géographique analysée se composait des territoires couverts par les services de santé. Les indicateurs sociaux ont été construits par analyse en composantes principales (ACP). Les cas ont été géocodés et le taux annuel de mortalité liée à la tuberculose a été calculé avec un lissage par la méthode bayésienne empirique locale, après quoi une régression linéaire multiple a été exécutée. L’existence d’une dépendance spatiale des résidus de régression a été confirmée par l’application du test Global I de Moran et l’application de modèles à effets spatiaux globaux, visant à identifier le meilleur critère de régression spatiale.

Résultats: Les taux de mortalité variaient de 0,00 à 2,8 décès pour 100 000 personnes par an. Trois indicateurs ont été construits dans l’ analyse ACP et désignés comme indicateurs de revenu, d’ inégalité sociale et d’ équité sociale. Dans la régression linéaire multiple, l’ indicateur d’ équité sociale était statistiquement significatif ($P < 0{,}0001$) mais avec une association négative, avec un R^2 ajusté de 28,36 % et une dépendance spatiale (Moran I = 0,21, $P = 0{,}003455$). Le meilleur modèle pour étudier la dépendance spatiale existante s’est avéré le modèle de latence spatiale.

Conclusions: L’amélioration des conditions sociales a entraîné une baisse de la mortalité liée à la tuberculose et ainsi consolidé la réalisation des Objectifs de développement durable. Une méthode de cartographie a également été appliquée. Transposable à d’autres scénarios dans le monde entier, elle utilise une approche distincte des travaux traditionnels puisqu’elle met l’accent sur l’équité sociale.

Translated from English version into French by Suzanne Assenat, through

Районы с доказательствами равенства и их прогресс с точки зрения смертности от туберкулеза, в эндемических муниципалитетах в Юго-Восточной Бразилии

Ямамура Меллина, Марселино Сантос Нето, Франсиско Кьяравалоти Нето, Луис Энрике Арройо, Антониу Карлос Рамос Виейра, Ана Анхелика Рего де Кейрос, Айлана де Соуза Бельчиора, Даниэль Талита дос Сантос, Юлиане де Алмейда Криспим, Йоне Карвальо Пинто, Ушоа Северина Алиса да Кошта, Регина Селиа Фиорати, Рикардо Александре Арсенсио

Аннотация

Общие сведения: В Бразилии, люди до сих пор болеют и умирают от туберкулеза (ТБ), и это можно объяснить значительными сложностями в справедливом распределении лечебных ресурсов для населения в целом. Целью было определить географические районы, которые показали прогресс с точки зрения справедливости (доходов, обучения в школе и городского размещения) и проверить его влияние на уровень смертности от туберкулеза в муниципалитете на юго-востоке Бразилии.

Методы: это экологическое исследование, учитывая ТБ как основную причину смертей, зарегистрированных в период между 2006 и 2013 по информационной системе смертности, и другие переменные, полученные через перепись Бразильского института географии и

статистики (2010). Географический район для анализа состоит из области охвата медицинскими услугами. Социальные показатели были построены через Анализ основных компонентов (АОК). Дела были геокодированны и ежегодный показатель смертности от туберкулеза был рассчитан со сглаживанием с помощью местного эмпирического байесовского метода. Затем была выполнена множественная линейная регрессия. Подтвердилось существование пространственной зависимости остатков посредством применения глобального теста Морана I и моделей с глобальными пространственными эффектами, чтобы определить лучший стандарт пространственных регрессий.

Результаты: смертности колебалась от 0,00 до 2,8 смертей на 100 000 человек в год. В АОК было построено три показателя: доходов, социального неравенства и социальной справедливости. В множественной линейной регрессии, показатель социальной справедливости был статистически значим ($P< 0.0001$) но имел негативные ассоциации, скорректированный R^2 28,36% и с пространственной зависимостью (Моран I = 0,21, $P= 0.003455$). Лучшей моделью для решения существующей пространственной зависимости была модель пространственного запаздывания.

Выводы: Более качественные социальные условия показали прогресс в сокращении смертности от туберкулеза, тем самым усиливая достижение целей устойчивого развития. Кроме того, была применена картография, которая может быть воспроизведена в других сценариях по всему миру, с применением масштаба, отличного от традиционного в том смысле, что акцент сделан на социальной справедливости.

Translated from English version into Russian by Elena Gheorghita, through

TRANSLATORS
WITHOUT BORDERS

Áreas con evidencia de equidad y su progreso en términos de mortalidad por tuberculosis, en un municipio endémico en el sureste de Brasil

Mellina Yamamura, Marcelino Santos Neto, Francisco Chiaravalloti Neto, Luiz Henrique Arroyo, Antônio Carlos Vieira Ramos, Ana Angélica Rêgo de Queiroz, Aylana de Souza Belchior, Danielle Talita dos Santos, Juliane de Almeida Crispim, Ione Carvalho Pinto, Severina Alice da Costa Uchoa, Regina Célia Fiorati, Ricardo Alexandre Arcêncio

Resumen

Antecedentes: En Brasil, todavía hay gente que enferma y muere por tuberculosis (TB). Esto se explica por el importante estancamiento en la igualdad de la distribución de recursos terapéuticos a la población en su conjunto. El objetivo consistió en identificar las áreas geográficas que han avanzado en cuestiones de igualdad (ingresos, escolarización y ocupación urbana) y analizar su efecto sobre la mortalidad por TB en un municipio del sureste de Brasil.

Métodos: Estudio ecológico que considera la TB como la causa básica de defunciones registradas entre 2006 y 2013 en el Sistema de Información de Mortalidad y otras variables obtenidas mediante el Censo Demográfico del Instituto Brasileño de Geografía y Estadística (2010). La zona geográfica objeto de análisis incluyó las zonas de cobertura de servicios sanitarios. Los indicadores sociales se han construido a través del Análisis de Componentes Principales (ACP). Los casos se geocodificaron y se calculó la tasa anual de mortalidad por TB con suavizado mediante el método Bayesiano empírico local. A continuación se realizó una regresión lineal múltiple. Se confirmó la existencia de una dependencia espacial de residuos a través de la aplicación del índice Moran Global (I) y la aplicación de los modelos con Efectos Espaciales Globales, para identificar la mejor norma de regresión espacial.

Resultados: Las tasas de mortalidad oscilaron entre 0,00 y 2,8 muertes por cada 100.000 personas, por año. En el ACP se construyeron tres indicadores y se asignaron como indicadores de ingresos, desigualdad social y equidad social. En la regresión lineal múltiple, el indicador de igualdad social resultó relevante a nivel estadístico (P<0,0001) pero tuvo una asociación negativa, una R^2 ajustada de 28,36% con dependencia espacial (Moran I= 0,21, P=0,003455). El mejor modelo para tratar con la dependencia espacial existente fue el Modelo de Rezago Espacial.

Conclusiones: La mejora de las condiciones sociales ha servido para reducir la mortalidad por TB, reforzando así el cumplimiento de los Objetivos de Desarrollo Sostenible. Además, también se aplicó cartografía, que se puede reproducir en otros escenarios en todo el mundo, con un alcance distinto de los proyectos que se han generado tradicionalmente porque hace hincapié en la igualdad social.

Translated from English version into Spanish by Denis Smyth, through

TRANSLATORS
WITHOUT BORDERS
